# Nucleolar GTP-Binding Protein 1-2 (NOG1-2) Interacts with Jasmonate-ZIMDomain Protein 9 (JAZ9) to Regulate Stomatal Aperture during Plant Immunity

**DOI:** 10.3390/ijms19071922

**Published:** 2018-06-30

**Authors:** Seonghee Lee, Clemencia M. Rojas, Sunhee Oh, Miyoung Kang, Swarup Roy Choudhury, Hee-Kyung Lee, Randy D. Allen, Sona Pandey, Kirankumar S. Mysore

**Affiliations:** 1 Noble Research Institute, LLC., Ardmore, OK 73401, USA; seonghee105@ufl.edu (S.L.); cr022@uark.edu (C.M.R.); soh@noble.org (S.O.); hklee@noble.org (H.-K.L.); 2 Gulf Coast Research and Education Center, Institute of Food and Agricultural Science, University of Florida, Balm, FL 33598, USA; 3 Department of Plant Pathology, University of Arkansas, Fayetteville, AR 72701, USA; 4 Institute for Agricultural Biosciences, Oklahoma State University, Ardmore, OK 73401, USA; mykang1021@gmail.com (M.K.); randy.allen@okstate.edu (R.D.A.); 5 Donald Danforth Plant Science Center, St. Louis, MO 63132, USA; srchoudhury@danforthcenter.org (S.R.C.); SPandey@danforthcenter.org (S.P.)

**Keywords:** Arabidopsis, guard cell, non-adapted pathogen, GTPase, coronatine, NOG1, Jasmonate-ZIM-domain protein 9 (JAZ9)

## Abstract

Plant defense responses at stomata and apoplast are the most important early events during plant–bacteria interactions. The key components of stomatal defense responses have not been fully characterized. A GTPase encoding gene, *NOG1-2*, which is required for stomatal innate immunity against bacterial pathogens, was recently identified. Functional studies in Arabidopsis revealed that NOG1-2 regulates guard cell signaling in response to biotic and abiotic stimulus through jasmonic acid (JA)- and abscisic acid (ABA)-mediated pathways. Interestingly, in this study, Jasmonate-ZIM-domain protein 9 (JAZ9) was identified to interact with NOG1-2 for the regulation of stomatal closure. Upon interaction, JAZ9 reduces GTPase activity of NOG1-2. We explored the role of NOG1-2 binding with JAZ9 for COI1-mediated JA signaling and hypothesized that its function may be closely linked to MYC2 transcription factor in the regulation of the JA-signaling cascade in stomatal defense against bacterial pathogens. Our study provides valuable information on the function of a small GTPase, NOG1-2, in guard cell signaling and early plant defense in response to bacterial pathogens.

## 1. Introduction

Plants are continually exposed to microorganisms present in the environment and, as a result, they have evolved intricate mechanisms to recognize them and mount a defense response against those that are potentially threatening. In contrast to fungal pathogens that are able to penetrate the plant epidermis, bacterial pathogens rely on wounds or natural openings to gain access into the plant [1]. One example of natural openings are the stomata, microscopic pores present on the plant surface that allow gas exchange between the plant and the atmosphere. Depending on the environmental and physiological conditions of the plant, the stomatal pores are opened or closed. This stomatal opening and closure is regulated by two guard cells that surround the pore and change their volume depending on the solute concentration, and consequently water amount [2]. When stomata are closed, they act as a physical barrier to bacterial pathogens. In contrast, open stomatal pores are an easy way of entry into the leaf apoplast, the site of bacterial multiplication [1].

In addition to environmental and physiological conditions, stomatal closure is also triggered by attempted penetration by pathogens as an inducible defense response. Plants recognize conserved surface features on pathogens called pathogen associated molecular patterns (PAMPs), which includes, among others, the flagellin-derived peptide flg22 and the bacterial lipopolysaccharide (LPS). PAMP recognition initiates a signal transduction cascade involving the activation of MAP kinases, production of reactive oxygen species (ROS) and changes in gene expression [3]. Although the presence of bacterial pathogens can cause PAMP-triggered stomatal closure, adapted plant pathogens are able to re-open stomata by means of virulence factors such as the phytotoxin coronatine (COR) [4]. COR mimics the plant hormone jasmonic acid-isoleucine (JA-Ile) in its chemical structure and function and consequently induces the expression of JA-Ile-inducible genes. JA-Ile-inducible genes are regulated by the transcription factor MYC2 [5] and the Jasmonate-ZIM domain (JAZ) proteins, a family of proteins that in addition to the ZIM domain also contain a Jas domain that is important for their interaction with transcription factors [6]. In the absence of JA-Ile, MYC2 interacts with a repressor complex formed by JAZ repressor proteins and the co-repressors Novel interactor of JAZ (NINJA) and Topless (TPL) to repress transcription of JA-Ile responsive genes [7]. In the presence of JA-Ile or COR, JAZ proteins bind the F-box protein Coronatine insensitive 1 (COI1), a subunit of the E3 ubiquitin ligase complex E3, and targets them for 26S proteasomal-mediated degradation [7,8].

The role of heterotrimeric G-proteins in guard cell signaling via the ABA pathway is well characterized [9]. The Gα subunit of the heterotrimer, which possesses the GTP-binding and GTPase activity, acts as a molecular switch to regulate diverse cellular processes by alternating between an active conformation (GTP-bound Gα and free Gβγ) and an inactive conformation (GDP-bound trimeric Gαβγ). Small monomeric G-proteins, also called small GTPases, are also widely conserved in eukaryotes and regulate many essential cellular processes [10]. We previously showed that two small GTPases, NOG1-1 and NOG1-2, have novel functions in plant immunity against bacterial pathogens and that NOG1-2 is a positive regulator of stomatal closure in response to both abiotic and biotic stresses [11].

In this study, we showed that NOG1-2 interacts with JAZ9 in Arabidopsis, suggesting that stomatal closure regulated by NOG1-2 occurs through the JA-mediated guard cell signaling network. This study together with our previous report [11], unravels the functional interplay between NOG1-2 and guard cell signaling that allow plants to fine-tune defense responses from stomata to apoplast against bacterial pathogens.

## 2. Results

### 2.1. NOG1-2 Functions in Guard Cell Signaling

**Figure 1:**
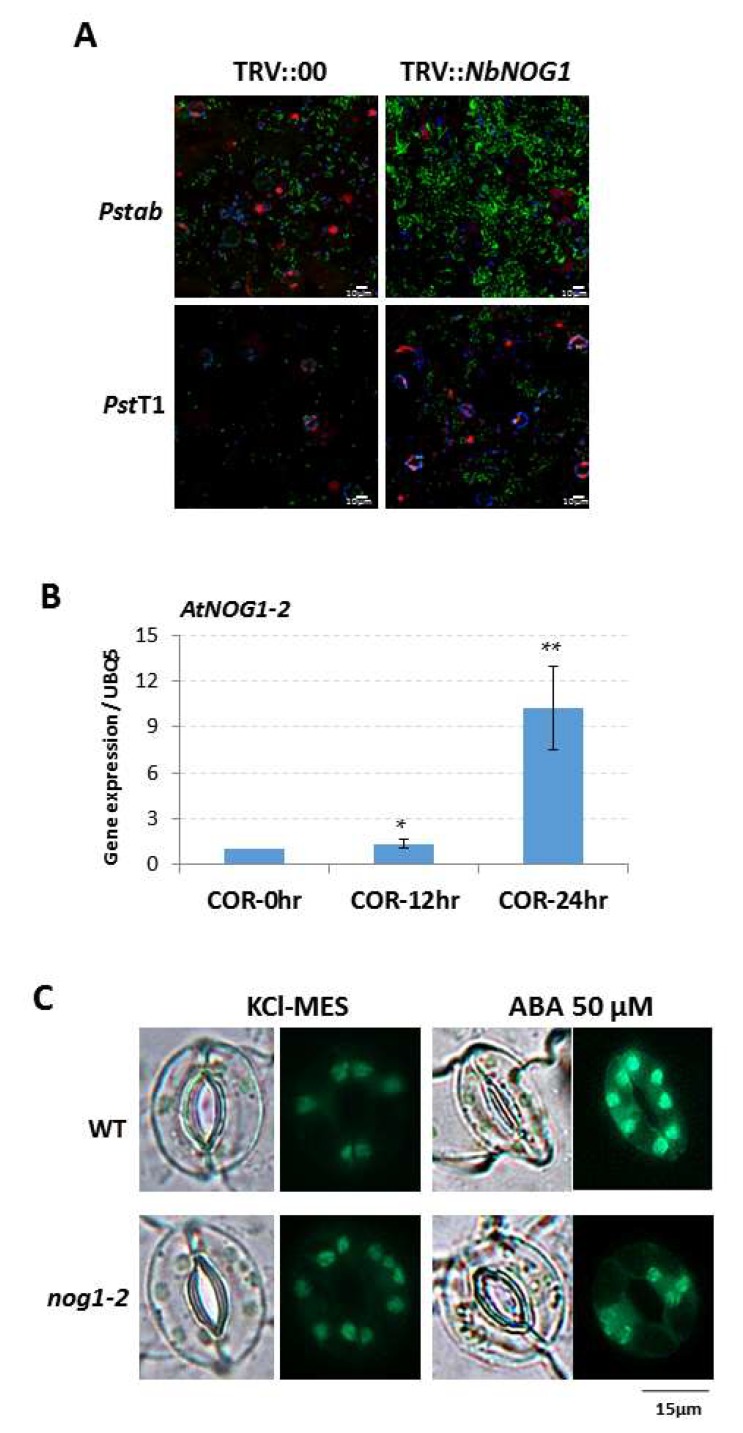
Functional role of NOG1-2 in guard cell signaling against bacterial pathogens and ABA. Epidermal peels from silenced- *N. benthamiana* plants (TRV::*NbNOG1*) and from non-silenced controls TRV::00 were incubated with the host pathogen *P*. *syringae* pv. *tabaci* (*Pstab*; 1 × 10^6^ CFU/mL) expressing *pDSK-GFP*uv [15] or the non-adapted pathogen *P*. *syringae* pv. *tomato* T1 (*Pst*; 1×10^6^ CFU/mL) expressing *pDSK-GFP*uv [15]. (**A**) Bacterial entry through stomata was observed by laser scanning confocal microscopy after 3 h of inoculation. Green: bacterial pathogens (*Pstab* or *Pst* T1) expressing *GFPuv*; Blue: auto-fluorescence of chloroplast; Red: nuclei. (**B**) *NOG1-2* expression was determined after treatment with COR (1 µM) in 4-week old seedlings grown on ½ MS media. Seedlings were dipped with the COR solution for 5 min and washed with distilled water. About five seedlings were obtained for qRT-PCR of *NOG1-2* at 12 and 24 h post treatment. The experiment was done with three biological replications and Student’s *t*-test was performed for statistical significance. Bars represent means ± standard deviation; * *p* < 0.05, ** *p* < 0.01 (Student’s *t*-test). *AtUBQ5* was used as an internal control. (**C**) Accumulation of reactive oxygen species (ROS) in guard cell is required for ABA-induced stomatal closure. Epidermal peels were incubated with DAF2-DA in KCl-MES buffer and ROS generation (green color) was monitored in wild-type and *nog1-2* 20 min after treatment with either ABA or KCl-MES (control).

In a previous study, we showed that Arabidopsis *nog1-2* mutant is defective in stomatal closure triggered by PAMPs and non-adapted pathogens. As a consequence of this defect, the *nog1-2* mutant supported higher rates of bacterial entry [11]. To confirm that the role of NOG1 is pathogen-triggered stomatal closure, we tested if silencing of *NbNOG1* in *Nicotiana benthamiana* inhibits stomatal closure and enables bacterial entry. Epidermal peels isolated from *NbNOG1*-silenced (TRV::*NbNOG1*) plants and incubated with the non-adapted pathogen, *Pseudomonas syringae* pv. tomato T1, expressing the green fluorescent protein (*pDSK-GFP_uv_*), showed significantly higher entry of bacteria into the apoplast than epidermal peels isolated from non-silenced control (TRV::00) plants (Figure 1A). A similar effect was observed after incubating epidermal peels with the adapted pathogen, *P. syringae* pv. tabaci (*pDSK-GFP_uv_*) (Figure 1A).

Further experiments were performed in Arabidopsis to gain insight into the stomatal function mediated by *NOG1-2*. The expression of *NOG1-2* was determined in response to the phytotoxin COR in Arabidopsis Col-0, given its properties altering stomatal function [12,13,14]. *NOG1-2* induction was observed 12 h after COR treatment. After 24 h of COR treatment, *NOG1-2* expression level was nearly 10-fold greater than control (0 h treatment; Figure 1B). Since COR effect is dependent on JA, the finding that *NOG1-2* is responsive to COR treatment, suggests that *NOG1-2* function is linked to JA signaling.

The importance of ABA in stomatal function prompted us to investigate the expression of *NOG1-2* in response to ABA in the stomatal guard cells. The publicly available eFP browser data (http://bar.utoronto.ca/efp2/Arabidopsis/Arabidopsis_eFPBrowser2.html) was used for searching gene expression of *NOG1-2* (Figure S1). The expression of *NOG1-2* in wild-type Col-0 showed approximately 3-fold induction in mesophyll cells after ABA treatment in comparison with water-treated controls, but only a slight induction of *NOG1-2* was observed in guard cells after ABA treatment in comparison with water treatment (Figure S1). Because stomatal closure is dependent on ABA-mediated accumulation of ROS [16], the accumulation of ROS was evaluated in the *nog1-2* mutant [11] in response to ABA. Epidermal peels of *nog1-2* and wild-type Col-0 plants were treated with KCl-MES or with 50 µM ABA and further incubated with the ROS responsive fluorescent sensor H2DCFDA [17]. Under UV illumination, fluorescence was significantly lower in the guard cells of *nog1-2* after treatment with ABA when compared to wild-type Col-0 (Figure 1C). This evidence points to the role of *NOG1-*2 in ABA-mediated stomatal closure. Taken together, these data indicate that NOG1-2 is likely to play a role in both ABA- and JA-mediated stomatal closures.

### 2.2. NOG1-2 Interacts with JAZ9, A Key Protein for Stomatal Closure

**Figure 2:**
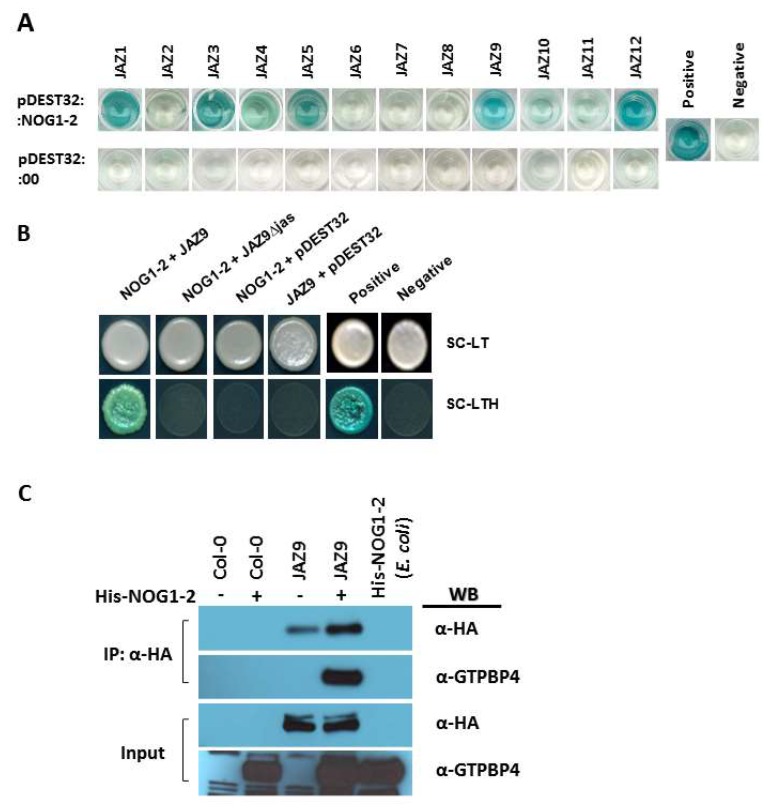
NOG1-2 interacts with JAZ9. **(A)** Full length NOG1-2 cloned into a bait plasmid pDEST32 was individually co-transformed with each full length JAZ protein (prey, pDEST22). The yeast clones were grown in quadrate dropout media (-leu, -try, -his, -ura) containing X-gal. A combination of pEXP32-Krev1 with pEXP22-RalGDS-wt was used as a positive control and pEXP32-Krev1 with pEXP22-RalGDS-m2 as a negative control (Thermo Scientific, Waltham, MA, USA). Image was taken seven days after incubation at 30 °C. **(B)** Yeast two-hybrid (Y2H) prey vector expressing full-length NOG1-2 was co-transformed in yeast with Y2H bait vector expressing JAZ9 or JAZ9 (Δjas) and plated on synthetic complete (SC) media lacking leucine, tryptophan, and histidine, and containing X-Gal to detect interaction by the development of blue-colored colonies. A combination of pEXP32-Krev1 with pEXP22-RalGDS-wt was used as a positive control and pEXP32-Krev1 with pEXP22-RalGDS-m2 as a negative control (Thermo Scientific). Empty bait vector, pDEST32, with either NOG1-2 or JAZ9 was used for autoactivation test. **(C)** Co-immunoprecipitation of NOG1-2 and JAZ9 in Arabidopsis. To examine the interaction between NOG1-2 and JAZ9, the purified His-tagged NOG1-2 protein in *Escherichia coli* was mixed with total protein extracts from Col-0 or HA-JAZ9 expressing transgenic plants and was later incubated with anti-HA agarose conjugating resin. Anti-GTPBP4 antibody was used to detect NOG1-2 protein. IP, immunoprecipitation; WB, western blot (**C**).

To gain a mechanistic insight into the NOG1-2-mediated regulation of stomatal closure, an Arabidopsis stress-induced yeast two-hybrid library was constructed (see Methods) and was screened using NOG1-2 as a bait to identify its potential interactors. A total of 13 putative NOG1-2 interacting proteins were identified (Table S1). Among the identified proteins, JAZ9 and PEN3 are associated with the function of stomatal closure in response to plant pathogens [18,19,20]. Since JAZ proteins are important for stomatal function against bacterial pathogens [21], we investigated the role of JAZ9 in NOG1-2-mediated stomatal closure. In addition, the potential interaction between NOG1-2 and other 12 JAZ proteins was tested. These data showed that NOG1-2 interacts with JAZ1, JAZ3, JAZ4, JAZ5, JAZ9, and JAZ12 (Figure 2A). Moreover, the interaction between NOG1-2 and JAZ9 was dependent on the presence of the JA-associated (Jas) domain, which is required for interactions with other major signaling proteins such as COI1 and MYC2 [22]. Full-length NOG1-2 (1-346 aa) strongly interacted with full-length JAZ9 (Figure 2B), however, a deletion of JA-associated (Jas) protein abolished the interaction with NOG1-2 (Figure 2B).

The interaction between NOG1-2 and JAZ9 was further validated using semi-in vivo co-immunoprecipitation in Arabidopsis. For this assay, protein extracts from transgenic Arabidopsis plants that overexpress *JAZ9* (fused to the hemagglutinin [HA] tag; [23]) and purified 6×-histidine (His)-tagged NOG1-2 protein from *Escherichia coli* were used. Anti-HA antibodies precipitated JAZ9-HA together with His-NOG1-2 (Figure 2D). Taken together, these data indicate that NOG1-2 interacts with JAZ9 both in vitro and in vivo and support the hypothesis that NOG1-2 is a component of the JAZ9 interactome, presumably for the purpose of JA-mediated stomatal closure.

### 2.3. JAZ9 Alters GTPase Activity of NOG1-2

**Figure 3:**
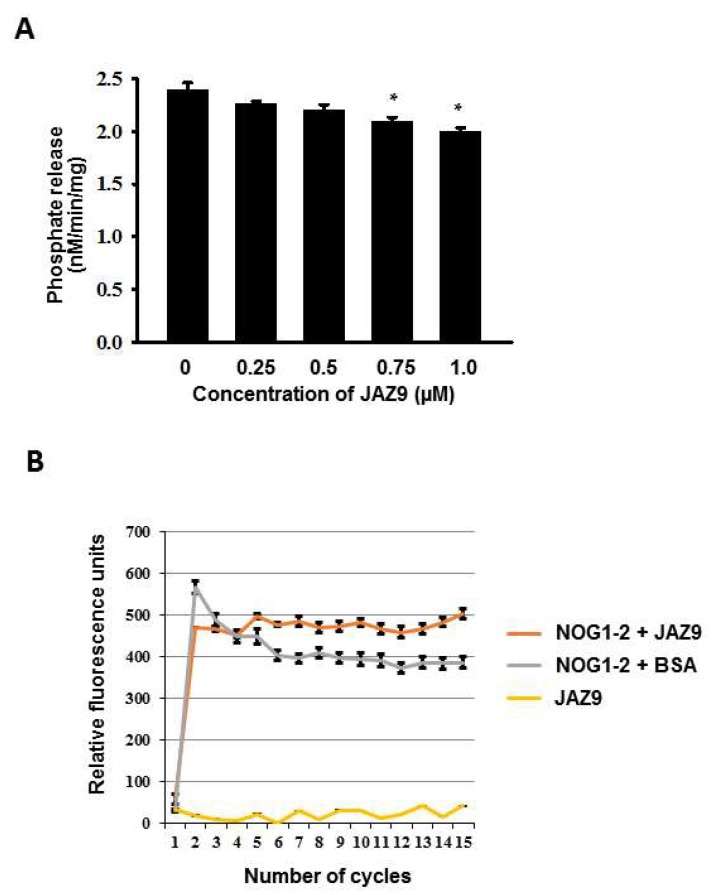
JAZ9 alters NOG1-2 GTPase activity. (**A**) The GTPase activity of NOG1-2 is reduced by JAZ9. Rate of Pi release due to the GTPase activity of NOG1-2 protein (1 µM) in the presence of varying concentrations of JAZ9. NOG1-2 protein (1 µM) was pre-loaded with GTP (1 mM) and incubated with or without different concentrations of JAZ9 protein (0.25–1μM). Phosphate (Pi) production was detected by ENZchek phosphate assay kit (Thermo Scientific). Experiments were repeated three times, and data were averaged. Error bars represent the mean ± standard error. Asterisks indicate statistically significant differences (* *p*< 0.05; Student’s *t*-test). (**B**) Real time fluorescence-based GTP-binding and hydrolysis assay. The upward slope represents GTP binding whereas the downward slope represents GTP hydrolysis by NOG1-2 protein. GTPase activity of NOG1-2 was reduced by JAZ9. BSA was used as a negative control.

NOG1-2 was previously shown to have GTPase activity in vitro [11]. To investigate the significance of the NOG1-2 and JAZ9 interaction, we assessed the GTPase activity of recombinant purified NOG1-2, in the presence of different concentrations of JAZ9, in a real time fluorescence-based GTP-binding/hydrolysis and a phosphate release assay [24,25] (Figure 3). NOG1-2 without JAZ9 released ~2.5 nM/min/mg of phosphate. However, the rate of GTP hydrolysis significantly decreased in the presence of increasing concentrations of JAZ9. With JAZ9 concentrations of 0.75 and 1 µM, phosphate release was reduced by about 20% when compared with that of NOG1-2 without JAZ9 (Figure 3A). A significant reduction in GTPase activity of NOG1-2 was also seen using the real time fluorescence-based GTP binding and hydrolysis of NOG1-2 in the presence of JAZ9 (Figure 3B).

### 2.4. NOG1-2 May Interfere with Interaction between JAZ9 and COI1

**Figure 4:**
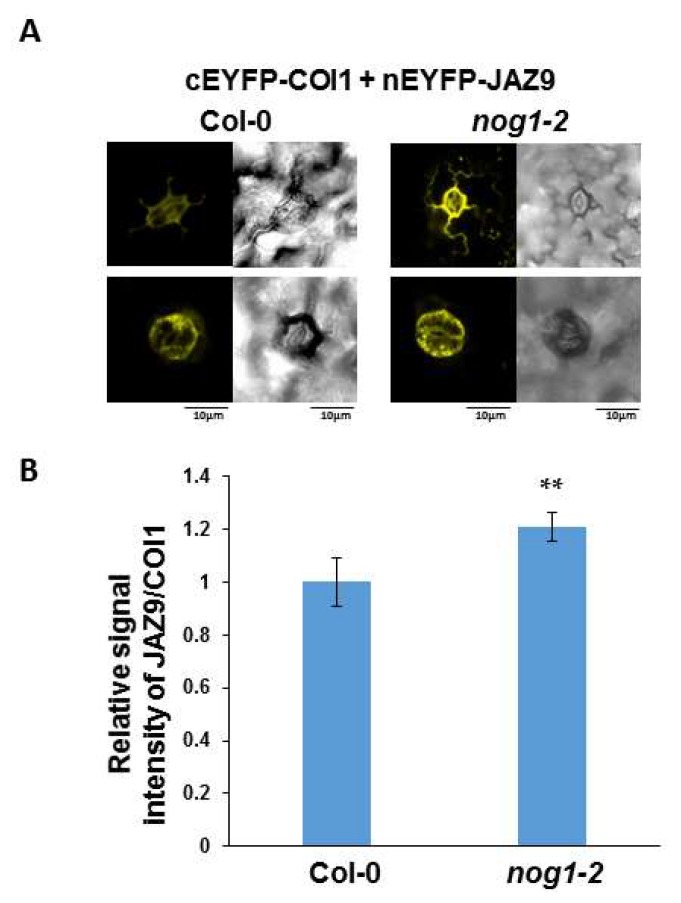
NOG1-2 may alter the interaction of JAZ9 with COI1. (**A**) BiFC in Arabidopsis was examined to test the interaction between JAZ9 and COI1. Seedlings were treated with COR (20 nM) and samples were collected after three hours for microscopy. JAZ9 fused to the N-terminal half of the EYFP was co-expressed with COI1 fused to the C-terminal half of EYFP in Col-0 or *nog1-2*. Reconstitution of the yellow fluorescence was observed in the guard cells of stomata. (**B**) Relative signal intensities of yellow fluorescence in the guard cells of Col-0 and the *nog1-2* mutant were quantified. Bars represent means ± standard deviation; ** *p* < 0.01 (Student’s *t*-test). Five seedlings were used for each experiment and the experiment was repeated three times.

Because JAZ9-COI1 interaction is important for stomatal function, we used BiFC in Arabidopsis to examine the role of NOG1-2 in this interaction. For that purpose, we transiently co-expressed *nEYFP-JAZ9*, containing a fusion of JAZ9 to the N-terminal half of EYFP, with *cEYFP-COI1*, containing a fusion of COI1 to the C-terminal half of EYFP in wild-type Col-0 and in the *nog1-2* mutant, using the Fast Agro-mediated Seedling Transformation (FAST) assay [26]. As previously reported [27,28], reconstitution of yellow fluorescence associated with the COR-induced JAZ9-COI1 interaction was observed in stomata in wild-type Col-0. Interestingly, the intensity of the fluorescent signal associated with the interaction was increased in the *nog1-2* mutant in comparison with wild-type Col-0 (Figure 4), indicating that in wild-type plants, NOG2-1 inhibits the interaction between JAZ9 and COI1. *P. syringae* effectors, such as COR and AvrB proteins were shown to induce COI1-JAZ9 interaction and degradation of multiple JAZ proteins during stomatal invasion [29]. This finding indicates that NOG1-2 can modulate COI1-JAZ9 interaction, leading to further regulation of stomatal closure against bacterial pathogens.

### 2.5. NOG1-2 Positively Regulates Signaling Pathways Related to Stomatal Function

**Figure 5:**
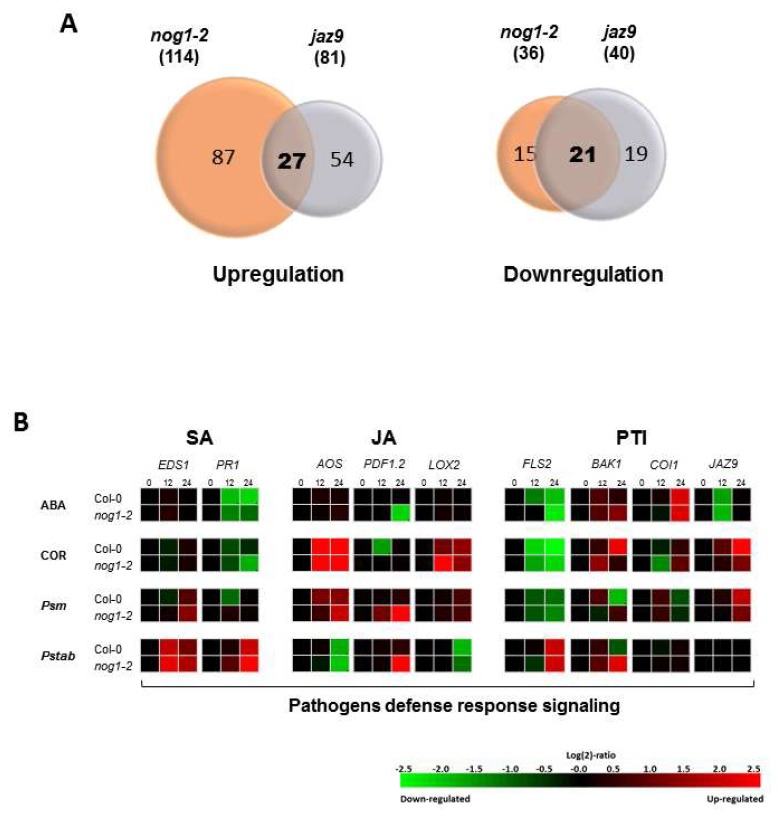
Gene expression profiling in Arabidopsis *nog1-2* and *jaz9* mutants. (**A**) Venn diagram showing the number of up and downregulated genes in *nog1-2* and *jaz9* mutants compared to wild-type (Col-0) with an overlap. Numbers in parenthesis are genes differentially expressed in a respective genotype. (**B**) Expression profiling of genes involved in SA and JA signaling pathways in Col-0 and *nog1-2*. Three-week-old Arabidopsis seedlings grown in MS medium were inoculated with ABA (100 nM), COR (20 nM), *P*. *syringae* pv. maculicola (*Psm*: 1 × 10^6^ CFU/mL) and *P*. *syringae* pv. tabaci (*Pstab*: 1×10^6^ CFU/mL) and samples were collected at 0, 12, and 24 h after inoculation for RNA extractions. qRT-PCR analysis was performed with three biological and three technical replications. *EDS1*: Enhanced Disease Susceptibility 1, *PR1*: Pathogenesis-Related Gene 1, *AOS*: Allene Oxide Synthase, *PDF1.2*: Plant Defensin 1.2, *LOX2*: Lipoxygenase, *FLS2*: Flagellin Sensitive 2, *BAK1*: BRI 1-Associated Receptor Kinase 1, *COI1*: Coronatine Insensitive 1, PTI: PAMP triggered immunity.

The finding that NOG1-2 function is linked to JAZ9 prompted us to compare the gene expression profiling of Arabidopsis *nog1-2* and *jaz9* mutants in comparison with that of wild-type Col-0 using Affymetrix GeneChip^®^ Arabidopsis Genome Array (Affymetrix, Santa Clara, CA, USA). A total of 114 and 81 genes were up-regulated, and 36 and 40 genes were downregulated, respectively, in *nog1-2* and *jaz9* mutants compared to Col-0 (Figure 5A, Tables S2 and S3). Several genes that were either upregulated or downregulated in both the *nog1-2* and *jaz9* mutants were identified. Specifically, 27 upregulated genes and 21 downregulated genes were found to be in common between these mutants (Figure 5A). Interestingly, several of the genes found to be downregulated in both *nog1-2* and the *jaz9* mutants are induced by ABA and drought stress, and consequently related to stomatal function (Figure S2). This finding indicates that NOG1-2 and JAZ9 may work together to induce ABA-related genes. Also, the microarray analysis showed that three *JAZ* genes, *JAZ1*, *JAZ5*, and *JAZ7*, were significantly upregulated in the *jaz9* mutant (Table S3). It is possible that JAZ9 function may be compensated for by JAZ1, JAZ5, and JAZ7 in Arabidopsis. JAZ1 and JAZ5 were also shown to interact with NOG1-2 in Arabidopsis (Figure 2A).

Because the differentially expressed genes identified by the microarray analysis in the *nog1-2* and *jaz9* mutants correspond to basal levels of expression, we decided to investigate the function of *NOG1-2* in the expression of genes associated with JA signaling: *AOS*, *PDF1.2*, *LOX2*, *COI1*, and *JAZ9* after treatment of Arabidopsis with ABA, COR, the adapted pathogen *P. syringae* pv. maculicola, and the non-adapted pathogen, *P. syringae* pv. tabaci. While the patterns of *AOS*, *LOX2*, and *JAZ9* gene expression were very similar between wild-type Col-0 and the *nog1-2* mutant, differences in the expression of *PDF1.2* and *COI1* were seen when comparing wild-type Col-0 with the *nog1-2* mutant. *PDF1.2* expression in the *nog1-2* mutant was downregulated in comparison with wild-type Col-0 after 24h of treatment with ABA. However, treatment with the non-adapted and adapted bacterial pathogens upregulated *PDF1.2* at 24 hpi in the *nog1-2* mutant in comparison to Col-0 (Figure 5B). *COI1* expression was downregulated in *nog1*-2, compared to Col-0, after 12 h treatment with COR (Figure 5B). We also showed that COR strongly induces *NOG1-2*, after 24 h treatment (Figure 1B). These results indicate that *NOG1-2* positively regulates *PDF1.2* in the presence of ABA, and *COI1* in the presence of COR. Taken together, our gene expression analyses indicate to us that NOG1-2 mediates cross-talk between the JA and ABA signaling pathways to mediate stomatal function during plant immunity.

## 3. Discussion

In a previous study, we showed that the small GTP-binding protein NOG1-2 was involved in the regulation of stomatal aperture and suggested the involvement of JA and ABA in this process [11]. This study was initiated to investigate the molecular mechanisms involved in NOG1-2-dependent stomatal regulation. Our results show that NOG1-2 interacts with JAZ9 (Figure 2). JAZ9 is a component of a repressor complex that regulates JA signaling by interfering with the activity of the transcriptional activator MYC2 [30]. Importantly, JAZ9 interaction with COI1 [14] relieves the repression by targeting the JAZ proteins to COI1-mediated proteasomal degradation [31]. We found that the GTPase activity of NOG1-2 was reduced by JAZ9 (Figure 3), indicating that NOG1-2–JAZ9 interaction could modulate downstream signaling required for stomatal closure in response to bacterial pathogens. It was also shown that the interaction between JAZ9 and COI1 was stronger in the *nog1-2* mutant background (Figure 4). This result implies that NOG1-2 interferes with the JAZ9–COI1 interaction, perhaps by competing with COI1. Since the interaction between NOG1-2 and JAZ9 occurs in the nuclei of guard cells [11], a likely scenario is that NOG1-2 displaces COI1 to modulate JA signaling that contributes to stomatal closure.

Our microarray analysis of *nog1-2* and *jaz9* mutants together with their gene expression data obtained from GENEVESTIGATOR (https://genevestigator.com/gv/), showing that NOG1-2 and JAZ9 are co-regulators of a core set of genes involved in ABA and abiotic stresses (Figure S2), indicates that JAZ proteins may also be connected with ABA-related pathways [32]. Thus, it appears that NOG1-2 integrates signals between JA and ABA pathways to achieve stomatal closure during biotic and abiotic stresses. Evidence for this hypothesis comes from our finding that NOG1-2 is important for the induction of JA-related genes after treatment with COR and ABA (Figure 5). While we showed that NOG1-2 positively regulates expression of genes involved in guard cell signaling, further research is needed to fully demonstrate that interaction between NOG1-2 and JAZ9 activates JA and ABA signaling pathways involved in stomatal function. Recently, proteomics approach reveals the functional role of JAZ7 in Arabidopsis defense response against bacterial pathogen and possible stomatal defense [33]. Similar proteomic analyses can be performed to investigate the functions for NOG1-2 and JAZ9.

*JAZ1*, *JAZ5*, and *JAZ7* were significantly upregulated in the *jaz9* mutant (Table S3), and yeast two-hybrid assay showed that JAZ1 and JAZ5 interact with NOG1-2 (Figure 2A). These results indicate that in the absence of JAZ9, JAZ1, JAZ5, and possibly JAZ7 may have a compensatory effect, consistent with earlier observation that JAZ proteins are functionally redundant [34]. *JAZ1*, *JAZ5*, *JAZ6*, *JAZ7*, and *JAZ9* are strongly induced by JA [31], indicating their functional relationship within the JA signaling pathway. Other studies also demonstrated that JAZ1, JAZ5, and JAZ9 interact with MYC2 [35,36].

MYC2, a key component of the JA signaling pathway, induces JA-responsive genes and JAZ proteins reduce its activity [37,38]. MYC2 has been shown to be phosphorylated by mitogen-activated protein kinase 6 (MPK6) in the regulation of seedling development and photomorphogenesis [39]. A proteomics study of guard cell proteins showed that MPK4, MPK9, MPK12, and MKK2 proteins are present in Arabidopsis guard cells [40]. Arabidopsis mutants with lesions in both MPK9 and MPK12 show reduced ABA promotion of stomatal closure and enhanced transpiration water loss in the leaves [41]. MPK9 and MPK12 show functional overlap in ABA and JA signaling [41,42]. It has been suggested that JAZ function is regulated by phosphorylation, but it has not been shown that phosphorylation is associated with COI1–JAZ interaction. In *N*. *benthamiana*, however, the JAZ3 homolog, NbPPS3, can be phosphorylated by MPKs to regulate plant cell death [43]. Arabidopsis JAZ12 was also reported to have phosphopeptides, using the PhosPhAt database [44]. JAZ13 contains a site for phosphorylation but the physiological significance of JAZ phosphorylation remains to be addressed. It would be interesting to investigate if any MAP kinases can phosphorylate JAZ proteins and modulate their interactions with COI1 or MYC2 during the regulation of JA signaling. More importantly, it will be interesting to determine if any of the MAP kinases interact with and phosphorylate NOG1-2. The phosphorylation of small GTPases by kinases that could potentially enhance their GTPase activity has been reported [45,46]. Our gene expression analyses showed that NOG1-2 activates expression of ABA-mediated pathways and ABA-dependent stomatal closure is known to require MPK9 and MPK12 [41].

Interesting questions remain regarding the interaction between NOG1-2 and JAZ9. For example, does NOG1-2 interact with other components in the COI1-JAZ complex for stomatal defense? How does NOG1-2 and JAZ9 interaction affect COI1 and MYC2-mediated JA signaling required for stomatal closure? We speculate that MPKs may also play a role in NOG1-2-mediated stomatal closure in response to biotic and abiotic stimuli. This warrants further investigation of the role of NOG1-2 in stomatal regulation through ABA and JA signaling. Nevertheless, identification of NOG1-2’s interaction with JAZ9 and other components of the JAZ complexes represents an important step toward a better understanding of the role of stomata during biotic and abiotic stresses.

## 4. Materials and Methods

### 4.1. Bacterial Entry Assay in Detached Leaf of NbNOG1-2 Silenced N. Benthamiana

Seedlings were conditioned to open stomata by placing plants under fluorescence light for at least 3 h. To determine bacterial entry via stomata, detached leaves from 3-week-old seedlings grown in ½ strength MS medium were floated on bacterial suspension: Adapted pathogen *P*. *syringae* pv. *tabaci* (*Pstab*; 1 × 10^6^ CFU/mL) expressing *pDSK-GFPuv* [46] or the non-adapted pathogen *P*. *syringae* pv. *tomato* T1 (*Pst*; 1 × 10^6^ CFU/mL) expressing *pDSK-GFPuv*. After 1 or 3 h incubation, leaf surfaces were sterilized using 10% bleach (Clorox), then observed with a fluorescence microscope. About five leaves were used for each assay, and the experiment was repeated at least three times.

### 4.2. Construction of the cDNA Library

To extract Arabidopsis RNA, the seedlings (Col-0) were grown in ½ MS for four weeks (12 h light/12 h dark condition, 28 °C). About eight seedlings were challenged with each treatment; adapted pathogen (*P. syringae* pv. maculicola), non-adapted pathogen (*P. syringae* pv. tabaci), ABA (100 nM), flg22 (1 µM), and coronatine (20 nM). For the pathogen inoculations, seedlings were inoculated with bacterial suspension (1 × 10^7^ CFU) for one minute and washed with distilled water. Seedlings were collected at 24 hpi for RNA extraction. Seedlings were treated with the solution of ABA, flg22, and COR for one minute and washed with distilled water, then collected at 12 hpi for RNA extraction. Total RNA was extracted using Qiagen RNeasy Mini Kit (Qiagen, Germantown, MD, USA) according to the manufacturer’s instructions. Full length cDNAs were synthesized using a CloneminerII cDNA Library Construction Kit (Thermo Scientific) according to the manufacturer’s instructions. The purified cDNA together with linearized pDEST22 cloning vector were co-transformed into MaV203 competent yeast cells.

### 4.3. Yeast Two-Hybrid Analysis

To identify NOG1-2 interacting proteins, we used Gal4 based yeast two-hybrid (Y2H) system as described by the manufacturer (Thermo Scientific). NOG1-2 full length (1–360 aa from Col-0) and truncated versions (1–150 aa) were initially cloned into pDONR207^®^ (Thermo Fisher Scientific) and subsequently transferred to the Y2H bait vector pDEST32 ^®^ (Thermo Scientific). The Y2H library was screened using the full-length NOG1-2 sequence as bait. NOG1-2 does not show autoactivation when tested against an empty vector on SD-Leu-Trp-His. Bait and prey (cDNA library) plasmids were co-transformed into the yeast cell and screened for the interaction following the manufactures protocol of ProQuest^™^ Two-Hybrid System with Gateway^™^ Technology (Thermo Scientific).

To examine interactions between fusion proteins, both bait (NOG1-2) and prey plasmids (Arabidopsis cDNA library) were co-transformed into a MaV203 yeast strain carrying three GAL4-inducible reporter genes (*lacZ*, *HIS3*, and *URA3*). Bait–prey interactions were selected on the synthetic dropout lacking Leu and Trp (SC-Leu-Trp). The yeast colony grown in SC-Leu-Trp was streaked on the medium lacking Leu, Trp, His, and Ura supplemented with 10 mM 3-AT (3-amino-1,2,4-triazole) with X-gal (20 µg/mL). Plasmids pEXP32/Krev1, pEXP22/RalGDS-m1, and pEXP22/RalGDS-m2 from Invitrogen were included as positive and negative controls, respectively, for interactions. Clones containing only prey were tested for autoactivation by growing them on SC-Leu-His with 10 mM 3-AT. For β-Galactosidase assays, yeast transformants were grown at 30 °C to mid-log phase (OD_660_ = 0.5–1.0) in the triple drop out liquid medium.

### 4.4. Bimolecular Fluorescence Complementation (Bifc) Analysis

The *JAZ9* and *COI1* genes were cloned into the pSITE destination and bimolecular fluorescence complementation (BiFC) vectors. Plant expression vectors used in this study were pSITE-BiFC-nEYFP-C1 and pSITE-BiFC-cEYFP-C1, purchased from The Arabidopsis Information Resource (TAIR). The full-length coding regions of the genes were fused in-frame with the fragments corresponding to the N (n-EYFP1-155) and C (c-EYFP156-239) termini of YFP driven by 2× *CaMV35S* promoter. The BiFC expression constructs pSITE-n-EYFP-target gene and pSITE-n-EYFP-target gene were transformed to disarmed *Agrobacterium tumafeciens* strain GV3101, and co-inoculated in Arabidopsis as described [26]. Each construct alone was infiltrated as a negative control. Four days after treatments, fluorescent images were observed with confocal laser microscopy (BioRad, Hercules, CA, USA). The intensities of BiFC signal were quantified using ImageJ software (https://imagej.nih.gov/ij/). 

### 4.5. In Vitro GTPase Activity Assay and Phosphate Release Assay

Real-time, fluorescence-based GTP-binding and GTP-hydrolysis assays were performed according to previously established protocols [47]. Briefly, the assays were performed at 25 °C in a 200 µL reaction volume of assay buffer (10 mM Tris, pH 8.0, and 10 mM MgCl_2_). For each assay, 200 nM of recombinant purified NOG1-2 protein was used with or without JAZ9 protein (200 nM). The reaction was started by the addition of a fluorescently labeled nucleotide; and the fluorescence (excitation 485 nm, emission 530 nm) was recorded using a fluorescence microplate reader (FLUOstar Optima, Cary, NC, USA). The amount of phosphate released due to the GTPase activity of NOG1-2 in the absence or presence of JAZ9 protein was measured using the ENZchek phosphate assay kit (Thermo Scientific, Waltham, MA) as described previously [11].

### 4.6. Semi-In Vivo Co-Immunoprecipitation

Arabidopsis seedlings (Col-0 and transgenic line expressing HA-tagged JAZ9 [27]) were homogenized in protein extraction buffer (50 mM Tris-HCl, pH 7.5, 75 mM NaCl, 0.2% Triton X-100, 5 mM EDTA, 5 mM EGTA,1 mM DTT, 100 µM MG132, 10 mM NaF, 2 mM Na_2_VO_4_, and 1% protease inhibitor cocktail). After protein extraction, 20 µg of purified His-NOG1-2 from *E. coli* was added to 1 mg of total protein from Col-0 and HA-JAZ9-expressing plants, then incubated overnight at 4 °C. The mixture was incubated for 3 h at 4 °C with anti-HA agarose conjugate resin. The precipitated samples were washed, then eluted by the addition of 2× SDS protein loading buffer and resolved by SDS-PAGE. This sample was further processed for western blot analysis using anti-HA and anti-GTPBP4 antibodies (https://www.genecards.org/cgi-bin/carddisp.pl?gene=GTPBP4).

### 4.7. Transcriptome Analysis of nog1-2 and jaz9 Using Arabidopsis Microarray

Arabidopsis seedlings were grown for seven days on ½ MS in controlled conditions with a 16 h light, 8 h dark cycle at 24 °C. Total RNA from three biological replicates of *nog1-2*, *jaz9*, and Col-0 leaves were isolated and purified by using the RNeasy MinElute Cleanup Kit (Qiagen, Germantown, MD, USA) and used for two-channel microarray. Experimental procedures of transcriptome analysis using Affymetrix ATH1 arrays were performed as described in the Affymetrix manual. For each sample, the excel files were exported from the Genechip Operating System (GCOS) program (Affymetrix) and normalized using the robust multichip average (RMA) protocol within R/Bioconductor to reduce technical variation. Only genes showing at least 2-fold up and downregulated expression relative to Col-0 in *nog1-2* and *jaz9* mutants were considered for analyses.

### 4.8. Quantitative Real-Time RT-PCR

All RNA samples were treated with DNase and used for cDNA synthesis. Quantification and purity of RNA and cDNA were determined using a nanodrop and agarose gel electrophoresis. The resulting cDNA was diluted 1:50 and 1 µL was used as a PCR template in a 10 µL reaction. For this qRT-PCR experiment, three biological replicates were included for each sample. The gene expression results in different RNA samples were normalized with the expression of internal control genes, *Actin2* and *UBQ9*, to ensure the equal amount of cDNA used for individual reactions. All qRT-PCRs were performed using ABI PRISM 7700 Sequence Detection System (Thermo Fisher Scientific, Waltham, MA, USA) and calculation was made according to the company manual.

## Supplementary Materials

Supplementary materials can be found at http://www.mdpi.com/1422-0067/19/7/1922/s1.
