# Supplementary material for: Nucleolar GTP-Binding Protein 1-2 (NOG1-2) Interacts with Jasmonate-ZIMDomain Protein 9 (JAZ9) to Regulate Stomatal Aperture during Plant Immunity
Source: Int J Mol Sci. 2018 Jun 30;19(7):1922. doi: 10.3390/ijms19071922 (PMC6073727; doi:10.3390/ijms19071922)
Supplement: Supplementary file 1 [file ijms-19-01922-s001.zip › Supplementary Files/Supplementary Figures.pptx]

## Slide 1
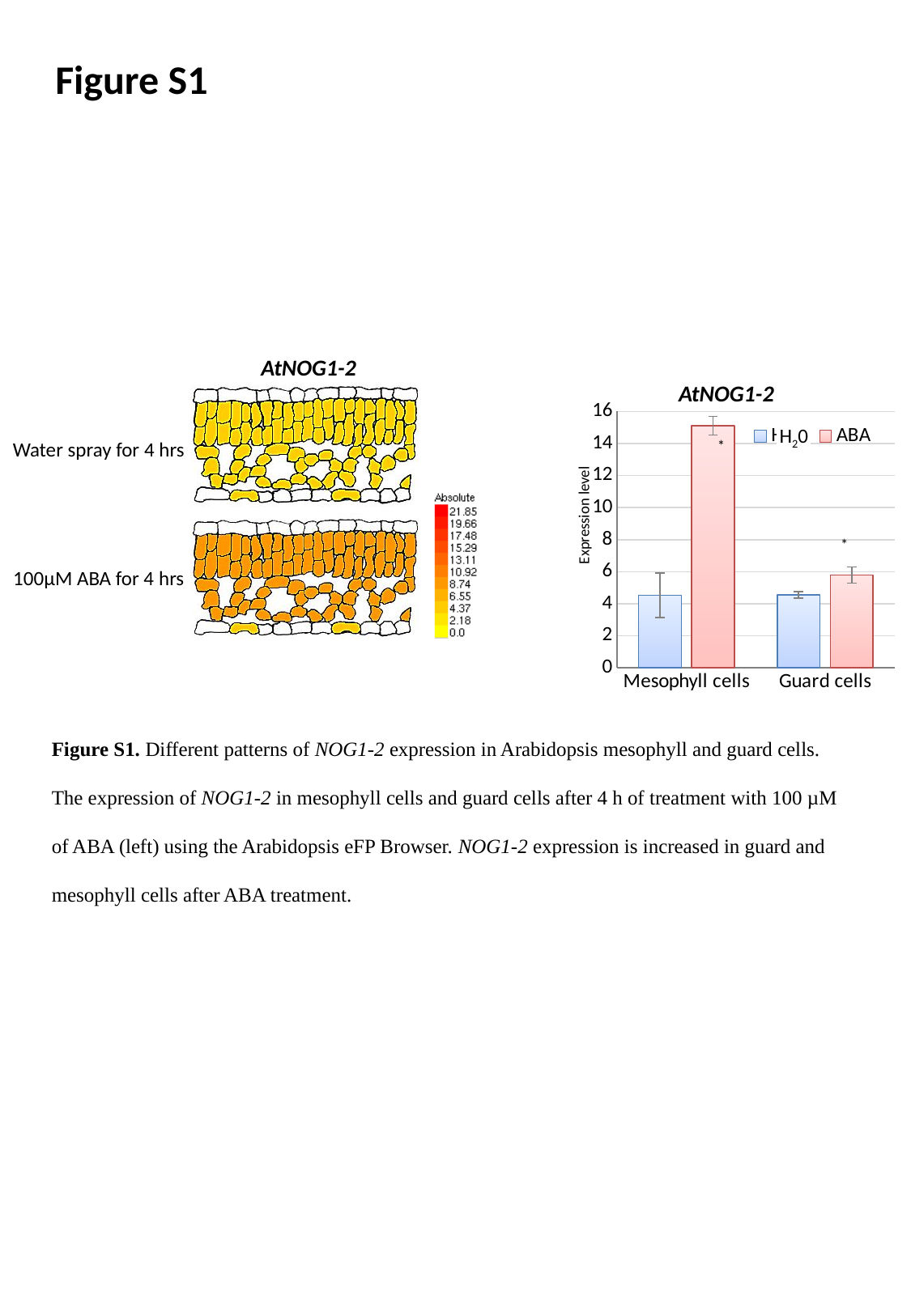

Figure S1
AtNOG1-2
AtNOG1-2
### Chart
| Category | H20 | ABA |
|---|---|---|
| Mesophyll cells | 4.52 | 15.12 |
| Guard cells | 4.56 | 5.79 |H20
*
Water spray for 4 hrs
Expression level
*
100µM ABA for 4 hrs
Figure S1. Different patterns of NOG1-2 expression in Arabidopsis mesophyll and guard cells.
The expression of NOG1-2 in mesophyll cells and guard cells after 4 h of treatment with 100 µM of ABA (left) using the Arabidopsis eFP Browser. NOG1-2 expression is increased in guard and mesophyll cells after ABA treatment.

## Slide 2
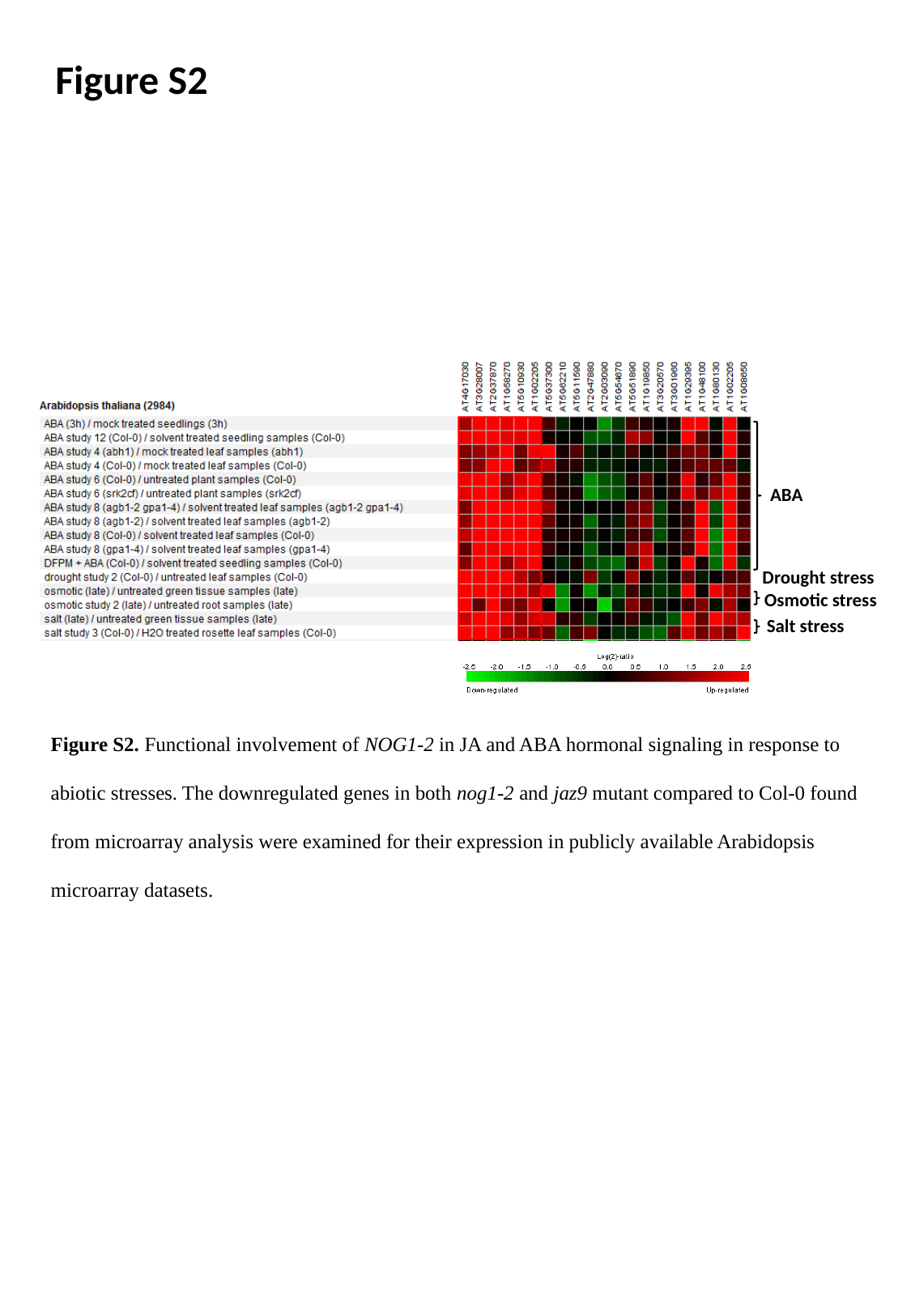

Figure S2
ABA
Drought stress
Osmotic stress
Salt stress
Figure S2. Functional involvement of NOG1-2 in JA and ABA hormonal signaling in response to abiotic stresses. The downregulated genes in both nog1-2 and jaz9 mutant compared to Col-0 found from microarray analysis were examined for their expression in publicly available Arabidopsis microarray datasets.
